# Valproic Acid-Induced Thrombocytopenia in Treatment-Resistant GABRB3 Genetic Epilepsy: A Case Report

**DOI:** 10.7759/cureus.57030

**Published:** 2024-03-27

**Authors:** Matthew Schuler, Ali Shammout, Maria Asif, Amy Mullikin

**Affiliations:** 1 Pediatrics, Western Michigan University Homer Stryker M.D. School of Medicine, Kalamazoo, USA; 2 Internal Medicine/Pediatrics, Western Michigan University Homer Stryker M.D. School of Medicine, Kalamazoo, USA

**Keywords:** treatment-resistant epilepsy, drug-induced thrombocytopenia, valproic acid, gabrb3, epilepsy treatment, epilepsy disorders

## Abstract

Valproic acid (VPA) is utilized in the management of a variety of seizure and mood disorders. A rare side effect of this medication is dose-dependent thrombocytopenia. In this case, we report a patient with a treatment-resistant epilepsy *GABRB3 *genetic variant phenotype who was admitted for sepsis and found to have significant thrombocytopenia with clinical manifestations of epistaxis and easy bruising, which was found to be due to VPA use rather than secondary to other clinical pathologies. The patient's clinical condition improved with supportive treatment including fluid rehydration. Platelet counts normalized after a transfusion and holding of her valproate. She experienced breakthrough seizures despite the initiation of diazepam. The decision was made to restart VPA per Neurology consult recommendations for better seizure control. She had no breakthrough seizures reported after restarting VPA in the hospital. This case highlights the importance of monitoring antiseizure medication side effects, especially in populations at higher risk due to treatment resistance.

## Introduction

Valproic acid (VPA) is used in the management of a variety of seizure and psychiatric disorders. While its mechanism is complex, it is most known for its inhibition of the degradation of GABA [1].

While VPA use is commonly associated with the side effects of hair loss, tremors, obesity, and insulin resistance, another rarer documented side effect of VPA use is thrombocytopenia. VPA-induced thrombocytopenia has been theorized to be secondary to dose-dependent bone marrow suppression [2]. In this case, we report a patient with a GABRB3 mutation contributing to treatment-resistant epilepsy relying on VPA for antiseizure management who initially presented with sepsis and was found to have profound thrombocytopenia with clinical manifestations of recurrent epistaxis and easy bruising. 

## Case presentation

**Figure 1:**
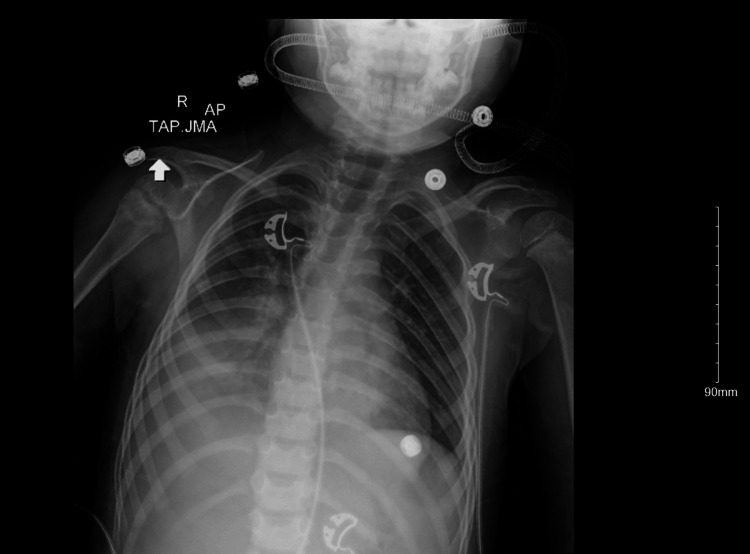
AP CXR at initial presentation showing basilar opacifications superimposed on moderate peribronchial wall thickening. No significant pleural process. Normal cardiac silhouette. AP: anterior-posterior view; CXR: chest X-ray

This patient is a four-year-old white female with a past medical history of epilepsy and GABRB3 mutation who initially presented to the emergency room with progressive difficulty breathing, fever, productive cough, and fatigue after a referral from urgent care due to oxygen desaturation. Upon arrival at the emergency department, the patient's respiratory disease panel was positive for influenza A, and the patient was diagnosed with sepsis. Initial chest X-ray showed moderate peribronchial wall thickening and basilar opacities bilaterally (Figure 1). Further workup revealed a platelet count of 14,000 platelets per microliter (mcL) on complete blood count. Further history revealed the patient had been experiencing ongoing episodes of easy bruisability and epistaxis with the patient having subsequent episodes of bleeding that was difficult to control after admission to the pediatric floor. The patient's mother noted that the patient's sister, who also has the mutation, also had low platelet levels while taking VPA.

At two years old, this patient was diagnosed with epilepsy after multiple episodes of tonic-clonic and absence seizures requiring hospitalization and was subsequently diagnosed with a GABRB3 mutation. The patient was initially started on daily valproate and clobazam for the management of her seizures without adequate seizure management. Subsequently, the patient was then initiated on a tapering schedule for her valproate to 250 mg twice daily and was simultaneously initiated on lamotrigine 20 mg once daily. After lamotrigine initiation, the patient's seizure control improved and remained under control. 

After admission, the patient's home medications were halted, and IV lorazepam was started as abortive therapy as needed for possible seizures. Serum VPA levels were found to be within the therapeutic range at 82.9 ug/mL. In addition to starting a high-flow nasal cannula for respiratory support, the patient was given a platelet transfusion that led to a sustainable increase in her platelet count. Serum antibody studies were negative for anti-GPIIB/3A antiplatelet antibodies. Pediatric Hematology was consulted and ruled out immune thrombocytopenic purpura (ITP) and thrombotic thrombocytopenic purpura (TTP) due to negative antibody screening, normal peripheral blood smear, and sustainable increase in platelet counts after platelet transfusion.

After consultation with her neurologist, the decision was made to restart her home medications but discontinue the valproate as this was most likely the cause of her thrombocytopenia paired with bone marrow suppression secondary to active viral infection. To prevent breakthrough seizures, the patient started oral diazepam via nasogastric (NG) tube and was briefly placed on an electroencephalogram (EEG) for monitoring which showed rare focal epileptiform discharges arising over the mid-temporal regions bilaterally and independently during sleep only without any seizure activity. However, the patient began having breakthrough seizures after EEG monitoring was discontinued. Once the patient's platelets normalized, the decision was made to restart the patient on her valproate, and the patient was then able to recover. Upon discharge, the patient had improved significantly and was advised to follow up outpatient with her neurologists for continued antiseizure medical management to prevent recurrence and with Hematology for monitoring of platelet levels.

## Discussion

VPA-induced thrombocytopenia has been documented to occur in 5-18% of patients with a dose-response relationship [3]. In a retrospective study, it was found that every 10 mcg/mL increase in serum VPA was associated with a decrease of 17 units/microliter in the platelet count with a significantly increased risk of thrombocytopenia in female patients with serum VPA levels over 80 μg/mL [4]. Additional risk factors for thrombocytopenia are female sex and lowered baseline platelet count, which can occur during acute viral infections [4-5]. In the presented patient case, it is important to note that bone marrow suppression secondary to acute viral illness most likely was a contributor to lowered baseline platelet levels in this patient.

Epilepsy usually presents early on in young patients with GABRB3 mutations and can pose a severe risk of intellectual disability, treatment resistance, encephalopathies, and hypotonia. In a large multicenter study, it was found that 58% of patients with GABRB3 mutations achieved seizure control with medication [6]. The high incidence of treatment resistance in GABRB3 mutations means that patients will often require multiple anti-epilepsy drug treatments to avoid breakthrough seizures [7]. Patients are then subject to multiple undesirable adverse effects from these medications. To our knowledge, there are limited case reports highlighting clinical sequelae and medication adverse effects associated with epilepsy secondary to GABRB3 mutations (Table 1).

**Table 1:** Summary of previous case reports on patients with epilepsy secondary to GABRB3 mutations highlighting treatment trials, treatments with best seizure control, clinical outcomes, and discussed medication adverse effects. References: [8-11] VPA: valproic acid

Author	Onset of seizures	Gender (M/F)	Treatment trials	Treatments with best control	Seizure control	Discussed adverse effects of medication
Zhang et al. (2017) [8]	3 days	F	VPA, levetiracetam, topiramate, ketogenic diet	Clonazepam	Complete cessation of seizures	None
Pavone et al. (2020) [9]	7 months	F	Stiripentol, phenobarbital	VPA, stiripentol, phenobarbital	Complete cessation	None
Bhelo et al. (2021) [10]	2 months	F	Phenobarbitone, levetiracetam	Clonazepam, pyridoxine	Decreased frequency, lost to follow-up	None
Papandreou et al. (2016) [11]	3 months	M	Vigabatrin, VPA, carbamazepine, levetiracetam, topiramate, ketogenic diet	None	Intractable epilepsy	None
Our patient	11 months	F	VPA, clobazam, diazepam, clonazepam	VPA, clobazam, lamotrigine	Complete cessation of seizures	Clinically manifested thrombocytopenia 12 months after the initiation of VPA

It is important to note that there are two main etiological theories of drug-induced thrombocytopenia (DIT): one being immune-mediated with peripheral platelet destruction and the other being caused by dose-dependent bone marrow suppression [12]. VPA-induced thrombocytopenia is currently believed to be secondary to dose-dependent bone marrow suppression [3]. In patients who experience DIT and subsequent bleeding side effects, treatment typically includes stopping the drug, administering platelet transfusions or managing bleeding, and counseling on future drug avoidance. The consequences of severe thrombocytopenia can be catastrophic, including sudden-onset internal bleeding and hemorrhagic stroke, with some cases documented to have resulted in death [6,13]. DIT is also very commonly misdiagnosed as ITP, which has a distinct treatment methodology from DIT, due to platelet transfusions having less efficacy in ITP [14]. 

The combination of side effects of antiseizure medications such as VPA with treatment-resistant epilepsy genetic phenotypes creates a perfect storm of complex patient management. After coordination with the widened healthcare team and discussion with the patient, the decision was ultimately made by the patient's family to restart her on VPA. DIT, especially in epileptic patients on VPA taking multiple medications, is important for providers to consider as a differential diagnosis when working up an acute case of thrombocytopenia.

## Conclusions

Epilepsy in children, especially those with underlying genetic mutations causing increased treatment resistance, can be very difficult to manage. Patients such as these often require multiple medications to manage seizures and prevent breakthroughs, exposing them to life-threatening risks. Valproate is a commonly used anti-epilepsy medication that, especially when used in conjunction with other medications, can lead to DIT exposing the patient to tremendous bleeding risk. It is important for providers to parse out the specific etiology of thrombocytopenia in these cases as management greatly varies depending on the proven etiology. Chronic management of these patients then also requires careful and informed balance of epilepsy control and adverse effect avoidance.
